# 0987. FAS activation alters tight junction proteins in pulmonary alveolar epithelial cells

**DOI:** 10.1186/2197-425X-2-S1-P72

**Published:** 2014-09-26

**Authors:** R Herrero, F Puig, R Guillamat, L Prados, Y Rojas, A Artigas, A Esteban, JA Lorente

**Affiliations:** Servicio de Cuidados Intensivos, Hospital Universitario de Getafe, Getafe, Spain; CIBERES (CIBER Enfermedades Respiratorias), ISC III, Madrid, Spain; Area de Cuidados Críticos, Corporació Sanitária i Universitária Parc Taulì-UAB, Sabadell, Spain; Laboratorio de Análisis Clínicos, Hospital Universitario de Getafe, Getafe, Spain; Universidad Europea de Madrid, Madrid, Spain

## Introduction

Active soluble Fas ligand (sFasL) accumulates in lung fluid of patients with acute respiratory distress syndrome (ARDS), and causes apoptosis and inflammation in lung epithelial cells [1]. Alveolar epithelial damage induced by Fas receptor activation results in protein-rich lung edema [2]. Dysfunction of the tight junction proteins may contribute to the formation of lung edema.

## Objectives

Determine whether sFasL increases protein permeability of the alveolar epithelium by mechanisms involving disruption of the tight junction proteins in ARDS.

## Methods

Primary human pulmonary alveolar epithelial cells were cultured in permeable transwell chambers. After reaching maximal confluency, the cells were incubated for 0.5, 1, 2 or 4 h with medium with or without human recombinant sFasL (rh-sFasL). Protein permeability of the cell monolayer was measured by using fluorescein-labeled albumin (FITC-Albumin). C56BL/6 wild-type mice and *lpr* (Fas deficient) mice were treated with an intratracheal dose of rh-sFasL (25 ng/g b.w.) or PBS, and the lungs were studied 16 h later. We performed immunofluorescence double staining for the detection of tight junction proteins (ZO-1 and Occludin) and apoptosis (Terminal Transferase dUTP Nick End Labeling assay).

## Results

In vitro, human sFasL increased protein permeability of the alveolar epithelial cell monolayer (medium only: 17.17 ± 2.4% vs rh-sFasL: 28.0 ± 3.6%, means ± SD, p< 0.05, t-test), altered the distribution of the tight junction proteins ZO-1 and Occludin, and induced apoptosis. In vivo, intratracheal instillation of rh-sFasL, which increases pulmonary protein permeability in wild-type but not in *lpr* mice, altered the distribution of ZO-1 and Occludin, and induced apoptosis in cells of the alveolar walls only in wild-type but not in *lpr* mice.

## Conclusions

Activation of the Fas/FasL system increased protein permeability of the pulmonary alveolar epithelium *in vitro* and *in vivo*. This increased permeability was associated with disruption of tight junctions and apoptosis. These results provide a mechanism that could be targeted for the prevention of lung edema in ARDS.

## References

[1] Matute-Bello G (1999). Soluble Fas ligand induces epithelial cell apoptosis in humans with acute lung injury (ARDS). J Immunol.

[2] Herrero R (2011). The biological activity of FasL in human and mouse lungs is determined by the structure of its stalk region. J Clin Invest.

